# Genetic algorithm with logistic regression for prediction of progression to Alzheimer's disease

**DOI:** 10.1186/1471-2105-15-S16-S11

**Published:** 2014-12-08

**Authors:** Piers Johnson, Luke Vandewater, William Wilson, Paul Maruff, Greg Savage, Petra Graham, Lance S Macaulay, Kathryn A Ellis, Cassandra Szoeke, Ralph N Martins, Christopher C Rowe, Colin L Masters, David Ames, Ping Zhang

**Affiliations:** 1 Digital Productivity Flagship, CSIRO, Marsfield, NSW, Australia; 2 The Florey Institute of Neuroscience and Mental Health, Parkville, Victoria, Australia; 3 CogState Ltd, Melbourne, VIC, Australia; 4 ARC Centre of Excellence in Cognition and its Disorders, and Department of Psychology, Macquarie University, Sydney, Australia; 5 Department of Statistics, Faculty of Science, Macquarie University, Sydney, NSW, Australia; 6 Food and Nutrition Flagship, CSIRO, Parkville, VIC, Australia; 7 Academic Unit for Psychiatry of Old Age, Department of Psychiatry, The University of Melbourne, VIC, Australia; 8 Mental Health Research Institute, Melbourne, VIC, Australia; 9 School of Exercise Biomedical and Health Sciences, Edith Cowan University, Perth, WA, Australia; 10 Sir James McCusker Alzheimer's Disease Research Unit, Perth, WA, Australia; 11 Department of Nuclear Medicine & Centre for PET, Austin Health, Melbourne, VIC, Australia; 12 Department of Medicine, University of Melbourne, VIC, Australia; 13 Department of Pathology, The University of Melbourne, VIC, Australia; 14 National Ageing Research Institute, Melbourne, VIC, Australia; 15 CRC for Mental Health, Melbourne, VIC, Australia

## Abstract

**Background:**

Assessment of risk and early diagnosis of Alzheimer's disease (AD) is a key to its prevention or slowing the progression of the disease. Previous research on risk factors for AD typically utilizes statistical comparison tests or stepwise selection with regression models. Outcomes of these methods tend to emphasize single risk factors rather than a combination of risk factors. However, a combination of factors, rather than any one alone, is likely to affect disease development. Genetic algorithms (GA) can be useful and efficient for searching a combination of variables for the best achievement (eg. accuracy of diagnosis), especially when the search space is large, complex or poorly understood, as in the case in prediction of AD development.

**Results:**

Multiple sets of neuropsychological tests were identified by GA to best predict conversions between clinical categories, with a cross validated AUC (area under the ROC curve) of 0.90 for prediction of HC conversion to MCI/AD and 0.86 for MCI conversion to AD within 36 months.

**Conclusions:**

This study showed the potential of GA application in the neural science area. It demonstrated that the combination of a small set of variables is superior in performance than the use of all the single significant variables in the model for prediction of progression of disease. Variables more frequently selected by GA might be more important as part of the algorithm for prediction of disease development.

## Introduction

There is agreement that the identification of risk factors for AD is important for both the diagnosis and prognosis of the disease AD [1]. Diagnosis of AD can not be made by a single test and requires careful medical evaluation, including medical history, mental status testing, physical and neurological exam, blood test and braining imaging etc. To this end there now exist large prospective studies of healthy older adults at risk for AD due to their age. Studies such as the Australian Imaging Biomarkers and Lifestyle (AIBL) Study of Ageing [2,3], the Alzheimer's disease Neuroimaging Initiative (ADNI) study [4,5] or the Mayo Clinic Study of Aging [6,7] measure a multitude of putative cognitive, biological, neuroimaging and lifestyle measures repeatedly. They aim to identify measures, or combination of measures that provide the earliest and most accurate prediction of progression to clinically classified AD. While the results of these studies suggest that body fluid and neuroimaging biomarkers of amyloid have great promise for early detection of AD, neuropsychological assessments of cognitive function have provided measures that are excellent predictors of progression to dementia [8].

Prospective studies like AIBL and ADNI, typically apply multiple neuropsychological tests, each hypothesized to measure different aspects of cognitive function, to their patient groups in repeated assessments. The objective of this approach is to identify performance measures upon which impairment or its change over time indicate the presence of early AD [9-11]. However, the use of multiple neuropsychological tests, each of which can also yield multiple outcome measures, provides a substantial analytic challenge in identifying which of these measures, either by themselves or in combination with others, are most predictive of dementia.

Many studies have considered each measure from each neuropsychological test as independent and conducted multiple analyses investigating the predictive value of each, e.g. [12-14]. Other studies have sought to limit analyses by restricting the number of outcome measures per neuropsychological test [12]. Both of these approaches are problematic because the multiple analyses increase the potential for the identification of false positive relationships. Other approaches have sought to understand the extent to which the different measures from different tests are actually related to one another by combining different outcome measures into theoretically based composite scores or by deriving sets of weighted composite scores from factor analysis [12,9]. These approaches to combining data do reduce the number of variables used in statistical analyses and thereby reduce the potential for identification of false positive relationships. However, the use of theoretically derived composite scores might also reduce the accuracy of prediction through inclusion of non-informative measures into these composite scores. Furthermore, while factor analytic solutions may improve definition of latent cognitive traits in a data set, the identified factors often have very little theoretical utility in the context of AD models [12]. Therefore, there is a need for exploration of other methods for combining data from neuropsychological tests batteries.

Genetic algorithms (GA) are machine learning search techniques inspired by Darwinian evolutionary models. The advantage of GA over factor analytic and other such statistical models is that GA models can address problems for which there is no human expertise or where the problem seeking a solution is too complicated for expertise based approaches. GA can be applied to challenges which can be formulated as function optimization problems. This makes GA ideal for application to discrete combinatorial problems and mixed-integer problems [15]. Thus the GA approach is appropriate for finding solutions that require efficient searching of a subset of features to find combinations that are near optimal for solving high-dimensional classification problems, especially when the search space is large, complex or poorly understood. The identification of the measures from multiple neuropsychological tests optimal for classifying early AD can be construed as such a problem. Thus with GA, the outcome measures of neuropsychological tests can be considered as features and a classification goal that the optimal combination of the tests is to achieve can be represented by a fitness (objective) function. Therefore the GA may be useful in identifying which neuropsychological measure or combination of measures from the baseline assessment in the AIBL study. They are helpful in identification of individuals whose disease has progressed to meet clinical criteria for mild cognitive impairment or Alzheimer's disease 36 months later. The aim of this study was therefore to use GA to determine and quantify the predictive value of combinations of neuropsychological measures for prediction of progression to MCI or AD from a normal healthy classification, and for prediction of progression to AD from MCI.

## Methodology

**Figure 1:**
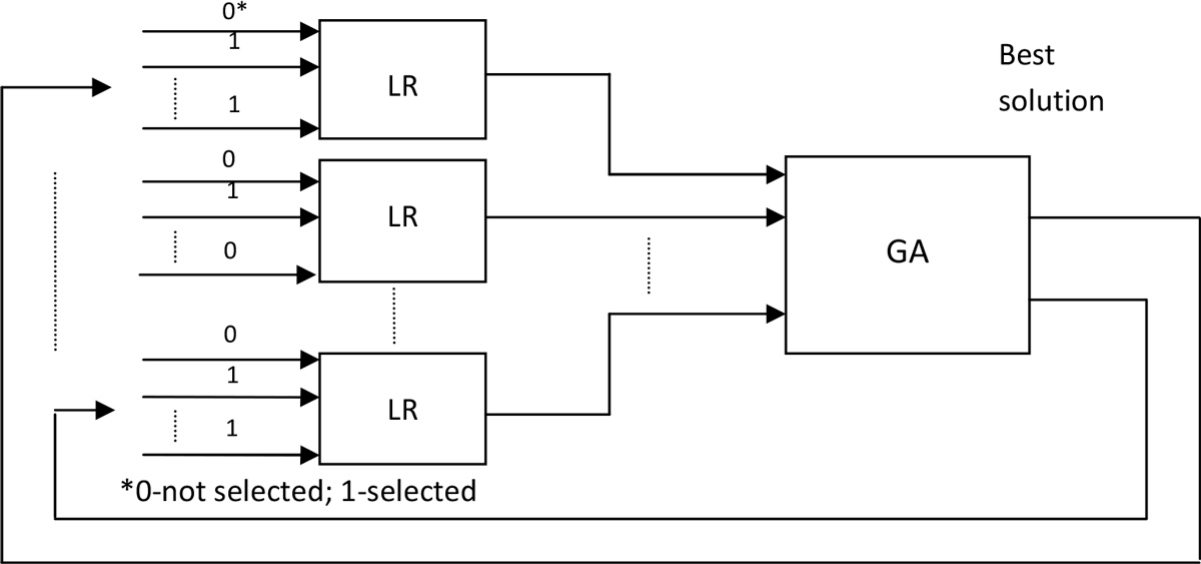
**The architecture of the system that combined the GA and LR in our study**. The search process involved three principal steps. LR was used with each set of features to make a predictive model for each instance within the possible solutions in GA. The subsequent cycles of the GA search find better solutions that replace less fit solutions found previously. This process is iteratively repeated until the goal solutions are found.

The goal of this study was to find the combinations of features that produce best-performing predictive models of AD early diagnosis and progression. In this study, a GA was used to select one or more sets of neuropsychological tests (features) which can predict AD progression with high accuracy and a logistic regression (LR) algorithm was used to build prediction models. The architecture of the algorithm and the system that combined GA and LR for the prediction of the AD status are shown in Figure 1. The features selected by the GA search were used as the input for LR, and the results from LR with different variable sets were used by the GA to perform an optimization and identify the best feature set. A similar method was used earlier in the study of heart disease [16,17].

### Data

A battery of neuropsychological and mood rating scales is given as part of the AIBL study. For this study data from the baseline assessment was used to predict the clinical classification of individuals at the 36 month assessment. The full assessment battery of tests comprised the:

• Mini-Mental State Examination (MMSE) [18]

• Logical Memory I and II (LM) - (WMS; Story 1 only) [19],

• California Verbal Learning Test - Second Edition (CVLT-II) [20],

• the Rey Complex Figure Test (RCFT) [21],

• D-KEFS verbal fluency (D-KEFS) [20],

• 30-item Boston Naming Test (BNT) [22],

• Digit Span and Digit Symbol-Coding subtests of the Wechsler Adult Intelligence Scale - Third Edition (WAIS-III) [23],

• the Stroop task (Victoria version) [24],

• Clock Drawing Test (CDT)[25],

• Wechsler Test of Adult Reading (WTAR) [26],

• Clinical Dementia Rating scale (CDR) [27], and

• Hospital Anxiety and Depression Scale (HADS) [28].

A comprehensive account of the cognitive battery of tests and the rationale behind the selection of individual tests was described in our previous paper [29]. The set of 37 cognitive and mood tests that covered the tests listed above and were used in our algorithm for selection of best feature subsets for prediction of AD progression are shown in Table 1. The Z scores of the tests, normed and age adjusted [29], were used in this study when available, otherwise raw scores were used.

**Table 1:** A complete set of neuropsychological tests used in this study.

Source	Cognitive test - feature	#	Source	Cognitive test - feature	#
MMSE	Mini Mental State Exam *	1	BNT	No Cue Australian	20

LM	Logical Memory I *	2		No Cue US	21

	Logical Memory II *	3	WAIS-III	Digit span	22

	Logical Memory Pass /Fail *	4		Digit symbol-Coding	23

CVLT-II	Total Learning (List A Trials 1-5)	5		UK Pred Full Score IQ *	24

	List A T6 Retention	6		US Pred Full Score IQ *	25

	List A 30 min Delayed Recall	7	Stroop	Dots time	26

	List A Recognition	8		Dots errs *	27

	List A False Positives	9		Words time	28

	Total Recognition d'	10		Words errs *	29

RCFT	Rey Complex Figure Copy	11		Colours time	30

	Rey Complex Figure Copy time	12		Colours errs *	31

	Rey Complex Figure 3 min delay	13		C/D	32

	Rey Complex Figure 30 min delay	14	CDT	Clock score *	33

	Rey Complex Figure Recog	15	WTAR	WTAR IQ score *	34

D-KEFS	Letter Fluency	16	CDRSoB	CDR Sum of Boxes *	35

	Category Fluency	17	HADS	Depression	36

	Category Switching Total	18		Anxiety	37

	Category Switching (switches)	19			

Each test is given a number (column #) that would be referred in the rest of the paper. * Z scores were not available or not applicable.

The AIBL population consisted of individuals aged 60 years or more who were classified as cognitively healthy, or who met clinical criteria for either the mild cognitive impairment (MCI) or AD. The diagnostic criteria and methods were previously described in [29]. After excluding the ones unavailable in 36 months(148), 13 deceased and 1 converted to vascular dementia from the total 797 HC cohort, the data set used for this research included 31 healthy controls (HC) who converted to either mild cognitive impairment (MCI) or to AD, and 604 who remained healthy over 36 months. These baseline data were used for building the models for prediction of HC conversion. The baseline MCI cases (47 converters and 30 non-converters at 36 months) were used to build the MCI conversion models, after excluding 49 unavailable ones in 36 months, 13 deceased and 3 converted to other dementias.

### Genetic algorithm

In GA, the potential solutions compete and mate with each other to produce increasingly fitter individuals over multiple generations. Each individual in the population (called genome or chromosome) represents a candidate solution to the problem. In our study genomes were represented by binary strings each encoding a set of features (variables) representing cognitive tests.

**Figure 2:**
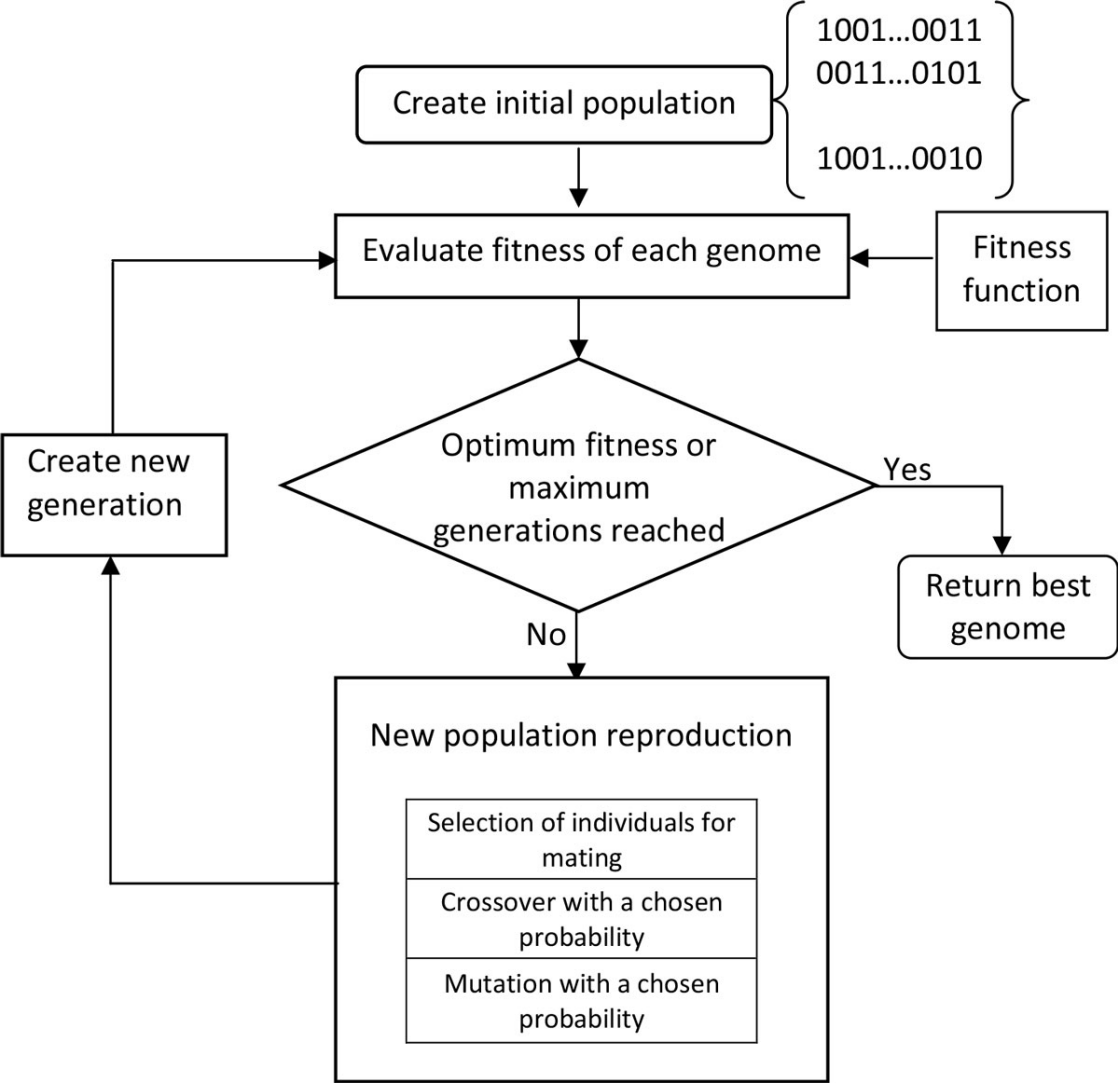
**Diagram representing the GA search**. Each solution within a generation is evaluated until the target solution is found. New generation of the population is produced using selection, crossover, and mutation operators that create new solutions. The "best" solution is returned when fitness function reaches target value

**Figure 3:**
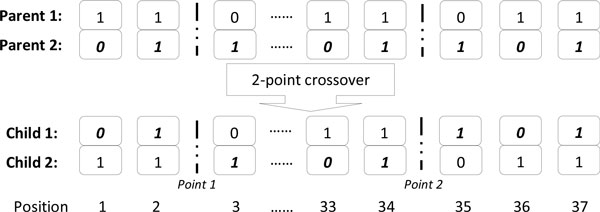
**An example of a 2-point crossover**. Two parent solutions exchange segments of their genomes. The swapped segments are from position 1 to Point 1, and from point 2 to position 37 of the genomes. Subsequently, the fitness of each solution is assessed, and if the fitness is improved the new "best" solution is added to the population of solutions.

A flow chart describing the overall GA used in this study is shown in Figure 2. Each individual in the population represents a candidate solution for feature subset selection problem from the set of 37 cognitive tests. The search space comprises 2^37 ^possible feature subsets. Each variable (cognitive test) in the GA was represented as a bit in the individual genome. A binary vector of length 37 represents each individual in the population of possible solutions. Each genome contained 37 bits, one bit for each feature/cognitive test. A bit value of zero (0) indicated that the corresponding feature was not selected, and a value one (1) indicated that the feature was selected. The initial population of potential solutions was randomly generated. Two-point crossover was chosen for reproduction of the next generation, noted as an optimal form of crossover for binary GAs in [30]. This involves selecting two random points within a genome of one parent and swapping the section between these exact two points with a second parent. For each crossover, two new individuals for the next generation were created, shown in Figure 3.

**Figure 4:**
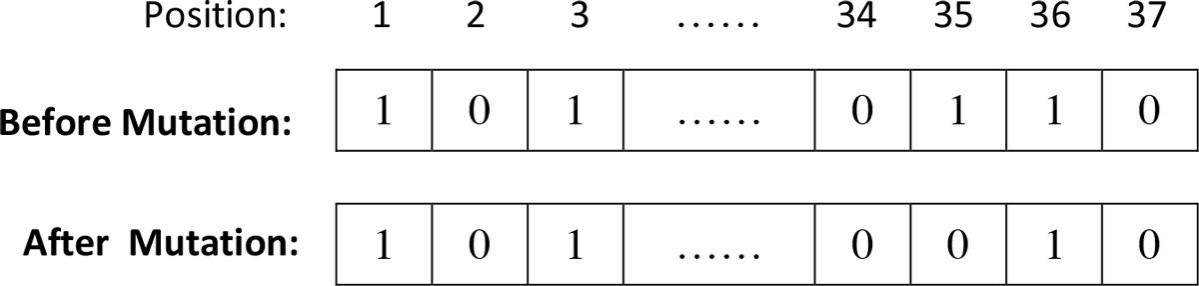
**Schematic showing a single point mutation**. In this example, position 35 was mutated from 1 to 0. The meaning of this transformation is that the corresponding feature (HADS Anxiety) was selected before the mutation and unselected after mutation.

The type of mutation chosen for the GA algorithm implementation was a single bit flip mutation, This was chosen to prevent large changes to the binary genome. Large changes are more likely to result in instability and most often result in a less fit solution after the mutation operation. The mutation operation was performed by randomly selecting a single bit on the genome and flipping it. An example of single point mutation is shown in Figure 4.

The selection of individuals for mating was done using the tournament selection. This method helps minimise early convergence of the algorithm. Early convergence of GA often produces poor solutions. The tournament selection allows for easily adjustment of selection pressure by selection of tournament size to further help tune the algorithm [31]. The tournament selection was performed by first taking a random sample from the population of the given tournament size. This individual "tour" was then used to select one parent by selecting the genome with the highest fitness. This sampling was then performed for the second parent, where the first parent could not be reselected. Crossover or mutation was then performed to these 2 parent individuals, with the chosen crossover and mutation rates. The two genomes produced by this process were then placed into the next generation. Elitism was used in the GA to ensure the best result was not lost from generation to generation. The fittest individual from the previous generation was copied and placed into the next generation replacing the least fit individual.

The best solutions, those that represent the fittest individuals, were defined as the sets of features that best classify the conversions from HC to MCI/AD or the conversions from MCI to AD, within 36 months, as assessed by the fitness function. To evaluate the fitness of any of the solutions, LR was used to build a prediction model using the given subset of features. The fitness of the genome was estimated by calculating the area under the curve of a receiver operating characteristic (ROC) produced by the LR model. Repeated five-fold balanced and stratified cross-validation [32] was used to assess the performance of the LR models.

The ROC curve is a graphical representation of the trade-off for every possible cut off (or a close approximation) between sensitivity (SE) and specificity (SP) [33]. It is calculated as an area under the ROC curve (AUC) that connects points (1-SP, SE) on the (x,y) plot for a range of values of SP that include SP = 0, SP = 1, and a number of values in between these two values. The plot provides a unified measure of the ability of a test to make correct classification being examined over all decision thresholds. The values of AUC = 0.5 indicate random guessing, AUC = 1 indicates perfect prediction, AUC = 0 is also perfect prediction but with class label switched. Furthermore the practical implications for majority of classification systems are that values of AUC>0.9 indicate excellent predictions, AUC>0.8 are good predictions, while AUC<0.7 indicate poor predictions [34].

### Logistic regression and fitness function

The main purpose of a fitness function is to evaluate the quality of each proposed solution. The function that evaluated the performance of models generated from any given variable set was based on the output result from the logistic regression model:

(1) $$\text{E}\left(\text{y}\right)=\frac{e^{t}}{1+e^{t}}$$

where y = 1, in this study, if the case converted from HC to MCI/AD or from MCI to AD in 36 months; y = 0, if the case didn't convert.

E(y) is the probability for conversion, that is for y = 1, in this study.

*t *is a linear function of explanatory variables that can be written as:

(2) $$t=\beta_{0}+\beta_{1}x_{1}+\beta_{2}x_{2}+\ldots +\beta_{k}x_{k}$$

where ${\text{x}}_{1},{\text{x}}_{2}\ldots {\text{x}}_{\text{k}}$ are the independent features (variables), and for this study they are the variables representing the cognitive test scores included in Table 1.

The AUC was used as the fitness function in the GA. This fitness function generally works well, but when in feature selection problems it tends to select larger feature sets. In this study, we aimed to select smaller variable sets that give similar classification results as the larger variable sets. Smaller variable sets have better utility because they are easier to understand and interpret. The collection of small sets of features is easier, faster and less costly, so it can be done more frequently. In clinical settings the collection of smaller variable sets has practical advantages since data collection is faster, cheaper, and easier for both patients and medical staff. To facilitate identification of small feature sets that are highly predictive, a penalty element was assigned to the larger variable sets in the fitness function:

(3) $$fitness\left(genome\right)=AUC+\rho -\frac{\rho l}{n}$$

where *ɭ *is the number of active variable's for the given genome, AUC is the area under the ROC curve, n is the total number of features and *ρ *is the compromise factor. More specifically, *ρ *is the offset of the ROC fitness that can be traded off to allow for selection of a smaller variable set.

### Implementation and cross-validation

The GA process was implemented in C programming language. The logistic regression models were built from the GLM function included in R [35] within the GA process, for each genome, the LR result was validated using repeated five-fold balanced and stratified cross-validation. Stratification was carried out by splitting the data into two sets, signifying positive and negative outcomes (e.g., cases converted to MCI from HC and the ones not converted). Each set was further divided at random into five equal folds, where fold size equality was enforced by the random removal of maximum one data point per fold. For each of the folds in turn, one part was used as validation data with the remaining parts used as training data to obtain an AUC value. The five AUC values from each of the validation data sets across the folds were averaged to obtain a single overall AUC value. The process of five-fold balanced and stratified cross-validation is repeated five times with different fold data combinations to mitigate the effects of bias in small observation datasets. The AUC value in the fitness function (3) is the average value of the five AUC values from the repeated cross-validation.

Each final LR model with the variable set selected from the GA was further analysed using Monte Carlo (MC) cross-validation. The data was randomly split into 80% for LR model training and 20% for validation. This was repeated for 1,000 runs and the AUC was averaged to give the final number. The repeated cross-validation technique employed by the fitness function was successful in minimising the bias encountered by non-repeated or fixed fold techniques. The higher accuracy of greater repetition averaging was not allowed due to high computational cost across the whole population over many generations. However, at the completion of the GA run, MC cross-validation on the final genome is less costly. This final cross-validation gave an AUC value that was used to compare the variable sets selected by each of the GA runs.

In this study, the GA was run multiple times for finding the best feature sets that predict conversion from HC to MCI/AD and conversion from MCI to AD respectively. A frequency chart was created from the features selected by all the GA runs to identify singular features that were selected more frequently than others.

### Simulations

In GA terminology, the specific runs are also called "experiments". This application has been developed for clinical settings, where an experiment often means wet-lab work or a clinical procedure. To prevent confusion, we call *in silico *experiments (GA runs) "simulations". GAs are by their nature very robust, however finding an optimal solution is not guaranteed when GA is used. To make the convergence of GA towards an optimal solution, we tried different search parameters.

The final values of search parameters were:

Population: 50

Mutation rate: 10%

Crossover rate: 90%

Tournament size: 2

Number of generation: 300

The final *ρ *value 0.085 in the fitness function was chosen to find relatively small sized variable sets that produce well-performing models for prediction of AD progression. The GA was run 50 times to find multiple sets of features that best (or close to best) predict the conversions from HC to MCI/AD or conversion from MCI to AD. The comparison of multiple runs allowed us to summarize frequencies of the features that appear in the best solutions selected by the GA search.

### Comparison with stepwise variable selection

To determine how well the GA performance comparing to more traditional statistical optimization techniques, a stepwise (SW) algorithm was deployed and the results were compared to those produced by the GA. The stepwise method started from a model that contained all the factors and a single factor was removed at each step until such point when removing any more factors would make the model worse. The actual stepwise function used was the stepAIC function from the MASS package [36]. The results were compared using paired t-tests.

### Building the final prediction models from the selected variable sets

To determine how the sets of features (variables) selected by the GA contribute to the classification models, logistic regression models were built using the complete set of available data with the GLM (General Linear Model) function from R. The models from representative variable sets from simulations define the *t *function in equation (2) for the HC to MCI/AD and MCI to AD conversion models, respectively.

## Results

### Prediction of conversion from HC to MCI or AD at 36 months

**Figure 5:**
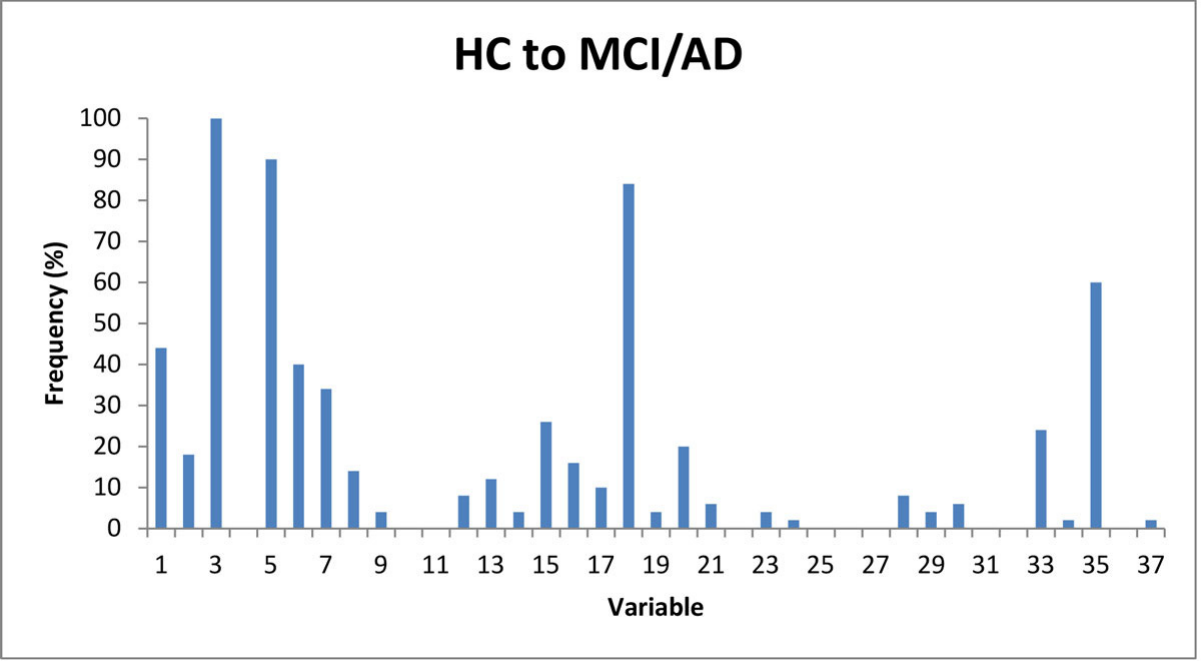
**Frequencies of the variables selected by GA, for prediction of conversion from HC to MCI or AD in 36 months**. Four features with frequencies greater than 50% (3, 5, 18 and 35; see Table 1 for details) were selected by the GA (in 50 runs).

Each run of the GA might find different 'best' set of features. Even for the same set of variables that were found, the AUC might be slightly different due to the differences in cross-validation sets associated with the individual runs. Initial inspection of feature combinations selected by the GA showed that the partitions produced equivalent prediction result (ROC). Some of the features were more consistently selected by the GA across different runs. Table 2 shows the results from multiple GA runs that predicted conversion of HC to MCI or HC to AD. The "Run #" is the GA run number (renumbered after 50 runs, ordered by the number of features in the feature sets from smallest to largest). The "MC_AUC" is the AUC value produced from the LR model using the GA selected variable set, validated by MC cross validation. The "Number of Variables" shows how many features were included in the feature set, with the corresponding features showed in the Column "Variables". Each feature is represented by a number matched to the cognitive test shown in Table 1. The series of numbers shown as "Variables" in Table 2 indicate the tests selected as the feature set. Variables 3, 5 and 18 (Logical Memory II, CVLT-II Total Learning and D-KEFS Category Switching Total) were frequently observed (>80%) in the final sets selected by the GA search (Figure 5). Together, these three features provide classification accuracy of AUC = 0.89. Furthermore, these three features are present in all selections that produced better results (AUC = 0.90 or AUC = 0.91) indicating they are critical for this task. Runs 7 and 8 have all five features that are present at frequency >40%, but their predictive performances are equal to performance of the three key features. Features 1 and 35 (Mini-Mental State Exam and CDR Sum of Boxes) are also frequently selected (>40%), however they appear to be redundant variables. To check whether there are any redundancies in the small size variable set (3,5,18) that performed well, we also checked the combinations of any 2 of the 3 variables, the prediction results from these 2-variable sets are all lower than using all 3 of the variables (Table 3). We checked the prediction performance from the models with each individual variable alone as an independent variable, there was not a single one variable that could perform better than the combination of variables 3, 5 and 18.

**Table 2:** GA results, HC to MCI or AD Conversion over 36 months.

Run#	MC_AUC	Number of Variables	Variables (see Table 1)	Run #	MC_AUC	Number of Variables	Variables (see Table 1)
**1**	0.89	3	3;5;18	**26**	0.89	7	3;5;6;15;18;21;35

**2**	0.90	4	1;3;5;18	**27**	0.88	7	3;5;6;13;15;18;35

**3**	0.88	4	3;5;6;30	**28**	0.89	7	3;5;7;18;20;33;35

**4**	0.86	4	3;6;16;33	**29**	0.88	7	3;5;6;16;18;20;35

**5**	0.89	5	1;3;5;6;18	**30**	0.89	7	3;5;7;15;18;33;35

**6**	0.90	5	1;3;5;18;29	**31**	0.89	7	3;5;7;18;20;35

**7**	0.89	5	1;3;5;18;35	**32**	0.87	7	3;5;6;13;15;35;37

**8**	0.89	5	1;3;5;18;35	**33**	0.88	7	3;5;7;15;18;21;35

**9**	0.89	5	1;3;5;18;28	**34**	0.89	7	2;3;5;7;8;18;35

**10**	0.89	5	3;5;18;22;35	**35**	0.90	7	3;5;6;16;17;18;35

**11**	0.89	5	3;5;15;18;35	**36**	0.88	7	1;3;5;7;18;33;28

**12**	0.89	5	3;5;6;18;33	**37**	0.88	7	1;3;5;7;12;19;33

**13**	0.87	5	1;3;6;18;33	**38**	0.89	7	2;3;5;9;15;18;35

**14**	0.88	5	1;3;6;18;20	**39**	0.89	8	1;2;3;5;6;15;18;35

**15**	0.88	5	2;3;5;30;35	**40**	0.88	8	1;3;5;7;15;18;20;35

**16**	0.87	5	1;3;6;9;16	**41**	0.89	8	2;3;5;7;18;20;33;35

**17**	0.89	6	3;5;6;18;20;35	**42**	0.90	8	1;3;5;12;13;14;17;18

**18**	0.89	6	3;5;6;18;21;35	**43**	0.88	8	1;2;3;5;7;15;18;35

**19**	0.89	6	1;3;5;7;8;18	**44**	0.89	8	1;3;5;7;8;18;20;33

**20**	0.89	6	3;5;6;15;18;35	**45**	0.89	8	1;3;5;13;14;18;33;35

**21**	0.89	6	3;5;6;18;35	**46**	0.88	8	3;7;12;18;24;30;35;36

**22**	0.89	6	3;5;6;18;33;28	**47**	0.91	9	1;3;5;7;8;13;16;17;18

**23**	0.88	6	2;3;5;6;15;28	**48**	0.89	9	2;3;5;7;8;16;18;20;35

**24**	0.88	6	3;5;7;15;19;23	**49**	0.90	9	1;3;5;7;8;13;18;33;29

**25**	0.90	7	1;3;5;16;17;18;35	**50**	0.90	11	1;3;5;6;8;12;16;17;18;20;35

The MC_AUC stands for AUC produced by the LR models for given sets of selected features. Variable sets with variable numbers ranging from 3 to 11 were found by the GA.

**Table 3:** AUC values from the LR models with each single variable or with any 2 of the most selected 3 features (variables).

Variable#	1	2	3	4	5	6	7	8	9	10	11	12	13	14	15	16	17	18	19
AUC	0.69	0.71	0.80	0.59	0.77	0.78	0.78	0.54	0.75	0.70	0.61	0.58	0.59	0.58	0.57	0.54	0.54	0.71	0.71

Variable#	20	21	22	23	24	25	26	27	28	29	30	31	32	33	34	35	36	37	

AUC	0.52	0.51	0.53	0.61	0.51	0.57	0.55	0.50	0.61	0.51	0.64	0.51	0.59	0.53	0.55	0.59	0.60	0.53	

	Variable set (with 2 variables)							5, 18				3, 5				3, 18			

	AUC							0.80				0.85				0.82			

The best value for a single variable was AUC = 0.8, the best value for two variables was AUC = 0.85, while the best value for selected set of small number of features was AUC = 0.90 (see Table 2).

Our results indicated that, for prediction of conversion from HC to MCI/AD the solution is dominated by the three features - 3, 5 and 18 that stand for Logical Memory II, CVLT-II Total Learning and D-KEFS Category Switching Total respectively. It appears that there is a redundancy in some tests. The inspection of results indicates that classification properties captured by feature 5 are most likely also captured by other variables, for example, 1, 20, and 33; properties captured by variable 18 are most likely captured by variables 6, 7, 15, and 35 etc; while feature 3 is essential. To make sure the solutions found by the GA are better than random chance selections, 100 sets of random variables for each corresponding length of feature set were selected and compared with the result from the GA selections. The results showed that the GA found better models than random chance (p < 10^-15^, t-test), see Table 4.

**Table 4:** Variable selection from GA for prediction of conversion from HC to MCI/AD, compared with random selections of features.

# of features	Average AUC (GA selected variable sets)	Average AUC (randomly selected variable sets)
3	0.89	0.69

4	0.88	0.71

5	0.89	0.74

6	0.89	0.75

7	0.89	0.77

8	0.89	0.77

9	0.90	0.77

11	0.90	0.79

The best performance is associated with sets comprising 3 to 11 features and can be classified as good-to excellent performance (AUC≈0.9). Random selection of features resulted in poor to borderline predictions (AUC<0.79).

### Prediction of conversion from MCI to AD

**Figure 6:**
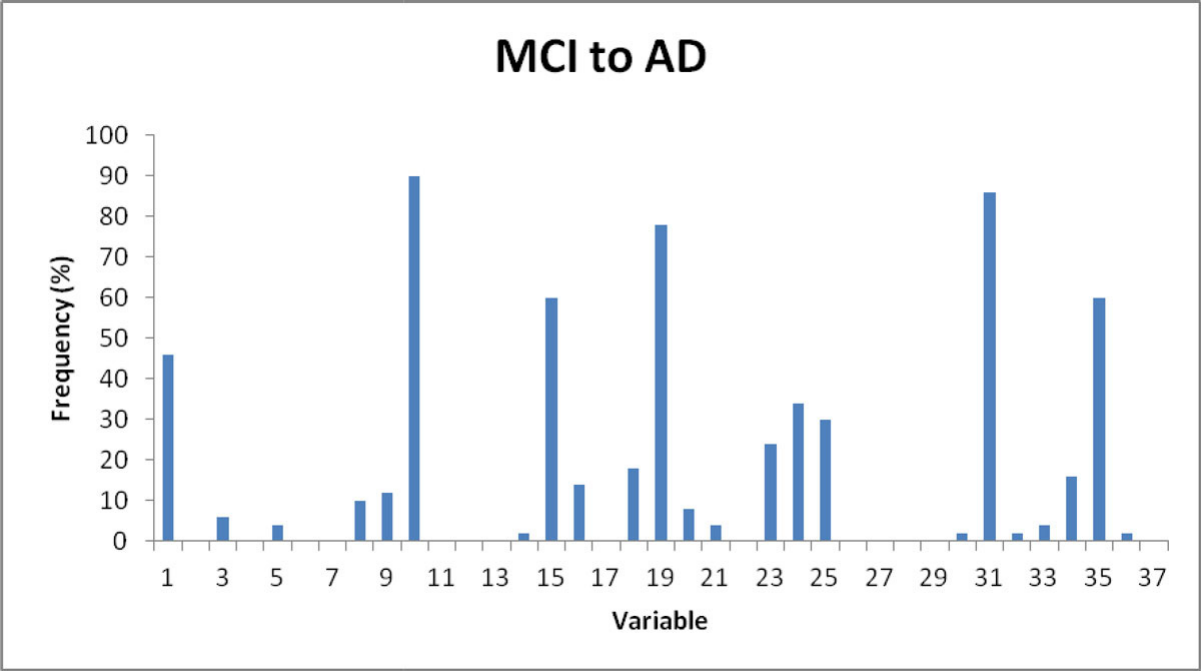
**Frequencies of variables selected by GA, for prediction of conversion from MCI to AD in 36 months**. Nine featuress were present in frequencies larger than 20%. The pattern of frequencies is dominated by five features, each present in more than 60% of feature sets.

Variables that occur frequently (>60%) in the feature sets include variables 10, 15, 19, 31 and 35 that stand for CVLT-II Total Recognition d', Rey Complex Figure Recognition, D-KEFS Category Switching (switches), Stroop Colours errs, and CDR Sum of Boxes, respectively. A number of other features occur with medium (>20%) frequency. The results from the 50 GA runs for predicting the conversion from MCI to AD are shown in Table 5. The frequencies of the variables being selected by GA in the final sets are shown in Figure 6. Notably the sets of features that make best predictors of conversion of MCI to AD do not overlap feature sets that are best predictors of conversion from HC to MCI/AD (only variables 1 and 35 appear frequently in both). The predictive performance of most MCI to AD conversion feature sets were AUC>0.85, with best predictive performance observed in predictors that use 4-8 features. The model with the 5 most frequently selected variables 10, 15, 19, 31, and 35 was also tested (run #12), providing an AUC value of 0.85. Run #14 with variables 10, 19, 24, 31, and 35 provided a similar AUC of 0.86, demonstrating that many combinations perform well (irrespective of individual variable selection frequency) and highlighting the power of the GA approach. Furthermore, the comparison with randomly selected feature sets showed superior performance of GA-selected features (p < 10^-15^, t-test), Table 6, indicating superior performance of the GA in this feature selection task.

**Table 5:** GA results, MCI to AD Conversion over 36 months.

Run #	MC_AUC	Number of Variables	Variables	Run #	MC_AUC	Number of Variables	Variables (see Table 1)
**1**	0.86	4	10;19;24;31	**26**	0.85	6	10;15;19;31;34;35

**2**	0.82	5	1;8;10;19;24	**27**	0.86	6	1;10;15;19;34;31

**3**	0.83	5	10;18;23;25;31	**28**	0.86	6	1;10;15;19;25;31

**4**	0.83	5	1;10;14;19;23	**29**	0.86	6	9;15;19;24;31;35

**5**	0.83	5	5;9;24;23;35	**30**	0.86	6	9;10;15;18;23;31

**6**	0.84	5	10;19;21;25;31	**31**	0.86	6	10;15;19;24;31;35

**7**	0.84	5	10;16;19;20;35	**32**	0.87	6	1;10;15;19;24;31

**8**	0.85	5	10;16;19;23;35	**33**	0.84	7	8;10;18;25;31;33;34

**9**	0.85	5	10;15;16;18;31	**34**	0.85	7	10;16;19;23;25;31;34

**10**	0.85	5	10;19;20;25;31	**35**	0.85	7	1;10;15;19;24;31;33

**11**	0.85	5	9;23;25;31;35	**36**	0.85	7	1;8;10;15;19;25;31

**12**	0.85	5	10;15;19;31;35	**37**	0.86	7	9;10;15;18;24;31;35

**13**	0.86	5	10;19;24;31;35	**38**	0.86	7	1;10;15;19;31;34;35

**14**	0.86	5	10;19;24;31;35	**39**	0.86	7	1;8;10;15;18;25;31

**15**	0.83	6	5;10;15;19;31;35	**40**	0.86	7	1;10;15;19;31;34;35

**16**	0.84	6	1;3;10;19;25;31	**41**	0.86	7	1;10;15;19;24;31;35

**17**	0.84	6	1;10;19;24;30;31	**42**	0.87	7	1;10;15;19;31;34;35

**18**	0.84	6	1;15;19;20;34;35	**43**	0.87	7	1;10;15;19;25;31;35

**19**	0.84	6	10;19;23;24;31;35	**44**	0.87	7	1;10;15;19;25;31;35

**20**	0.84	6	3;10;19;23;31;35	**45**	0.87	7	1;10;15;19;25;31;35

**21**	0.85	6	1;10;19;23;31;35	**46**	0.87	7	10;15;16;18;20;31;35

**22**	0.85	6	10;16;19;24;31;35	**47**	0.87	7	1;10;15;19;24;31;35

**23**	0.85	6	9;15;18;23;31;35	**48**	0.85	8	3;10;15;16;19;21;25;35

**24**	0.85	6	8;10;15;18;25;31	**49**	0.86	8	1;10;15;19;24;31;35;36

**25**	0.85	6	1;10;15;19;23;31	**50**	0.87	8	1;10;15;19;24;31;32;35

The MC_AUC is the accuracy of predictions measured by the AUC value. The best results involve feature sets with 4-8 variables, while longer solutions (more variables in the models) were rejected by the GA selection criteria (worse performance).

**Table 6:** Variable selection from GA for prediction of conversion from MCI to AD, compared with random selections of features.

# of features	Average AUC (GA selected variable sets)	Average AUC (randomly selected variable sets)
4	0.86	0.67

5	0.85	0.67

6	0.85	0.68

7	0.86	0.69

8	0.86	0.69

The best performance is associated with the feature sets of lengths 7 and 8, that can be classified as good performance (0.9>AUC>0.8). Random selection of features resulted in poor prediction (AUC<0.7).

### Comparison with stepwise variable selection

The comparative results from GA and the Stepwise Optimization with the constraint for sample size are shown in Table 7. For prediction of conversion from HC to MCI/AD, the performance of variable sets selected by GA and those selected by stepwise algorithm did not show significant difference. The result indicated that features 18 (Category Switching Total) and 19 (Category Switching (switches)) are very similar but feature 18 is preferred in most predictors. The feature sets selected by GA for prediction of conversion from MCI to AD were significantly better predictors than the features selected by stepwise algorithm (p = 0.002).

**Table 7:** Results from the models selected by GA and from the stepwise algorithm.

Case	Size	GA		Stepwise		P_value (t test)
		
		Variables	AUC	Variables	AUC	
HC to MCI	3	3;5;18	0.89	3;5;19	0.88	0.366
		
	4	1;3;5;18	0.90	3;5;8;18	0.89	
		
	5	1;3;5;18;29	0.90	3;5;8;17;18	0.89	
		
	6	1;3;5;7;8;18	0.89	3;5;8;16;17;18	0.90	
		
	7	3;5;6;16;17;18;35	0.90	3;5;8;9;16;17;18	0.90	
		
	8	1;3;5;12;13;14;17;18;35	0.90	3;5;8;9;16;17;18;33	0.91	
		
	9	1;3;5;7;8;13;16;17;18	0.91	3;5;8;9;16;17;18;33;25	0.90	
		
	11	1;3;5;6;8;12;16;17;18;20;35	0.90	1;3;5;8;9;16;17;18;25;33;34	0.91	

MCI to AD	4	10;19;24;31	0.86	10;16;19;35	0.83	0.002

	5	10;19;24;31;35	0.86	10;16;19;27;35	0.80	
		
	6	1;10;15;19;24;31	0.87	10;16;19;27;28;35	0.80	
		
	7	1;10;15;19;24;31;35	0.87	10;16;19;24;27;28;35	0.79	
		
	8	1;10;15;19;24;31;32;35	0.87	10;16;18;19;24;27;28;35	0.77	

No significant difference was observed for use of GA and stepwise algorithm for prediction of progression from HC to MCI/AD, while GA was superior in predicting progression from MCI to AD.

### Final LR prediction models built from selected variable sets

To check the contribution of each variable in the models, we built the models with the whole set of data using the selected variable sets from GA and stepwise selections. Models m1G, m2G, m3G and m4G (below) were built using the variables selected by GA, and m1S, m2S, m3S and m4S are the models built using the variables selected by the stepwise algorithm. ${\text{t}}_{\text{HCcon}}$ and ${\text{t}}_{\text{MCIcon}}$ represent the *t *functions in equation (2) for HC to MCI/AD and MCI to AD conversion. The $v_{i}$ are the explaining variables in the equation, representing variables from Table 1, for example, $v_{1}$ represents variable 1 in Table 1 (Mini-Mental State Exam). The p values for each variable in the models were from the z statistical tests against the null hypothesis that the true value of each coefficient is 0, and they are shown in Table 8.

**Table 8:** P values of each variable in the different models.

Conversion from HC to MCI/AD					Conversion from MCI to AD				
**Variables**	**Pvalues_m1G**	**Pvalues_m1S**	**Pvalues_m2G**	**Pvalues _m2S**	**Variables**	**Pvalues _m3G**	**Pvalues _m3S**	**Pvalues _m4G**	**Pvalues _m4S**

V1	0.07		**0.002**		V1			0.170	

V3	**<0.001**	**<0.001**	**<0.001**	**<0.001**	V10	**0.011**	**0.019**	0.056	**0.011**

V5	**0.003**	**<0.001**	**0.014**	**<0.001**	V15			**0.048**	

V7			**0.004**		V16		**0.046**		0.117

V8		**0.01**	0.251	**0.012**	V18				0.245

V9				**0.021**	V19	**0.009**	**0.006**	**0.008**	**0.026**

V13			0.139		V24	**0.043**		**0.034**	0.124

V16			0.109	0.087	V27		0.284		0.233

V17			**0.004**	**0.020**	V28				0.092

V18	**0.007**	**0.003**	**0.001**	**0.004**	V31	0.142		0.154	

V25				0.828	V32			0.549	

V33				0.063	V35	0.104	**0.014**	0.181	**0.033**

The results provide statistical support for our finding that variables 3, 5 and 18 dominate prediction of progression of HC to MCI/AD. The prediction of progression from MCI to AD consistently showed the importance of variable 19, and also of variables 10 and 35. These results indicate that for conversion of MCI to AD, the combinations of variables are more important than the contribution of individual variables.

(m1G) $${\text{t}}_{\text{HCcon}}=13.43-0.31v_{1}-0.27v_{3}-0.06v_{5}-0.18v_{18}$$

(m1S) $${\text{t}}_{\text{HCcon}}=6.001-0.28v_{3}-0.078v_{5}+0.74v_{8}-0.19v_{18}$$

(m2G) $${\text{t}}_{\text{HCcon}}=17.58-0.46v_{1}-0.293v_{3}-0.07v_{5}-0.31v_{7}+0.84v_{8}-0.29v_{13}-0.11v_{16}+0.25v_{17}-0.23v_{18}$$

(m2S) $${\text{t}}_{\text{HCcon}}=-3.98-0.27{\text{v}}_{3}-0.07{\text{v}}_{5}+0.82{\text{v}}_{8}+0.53{\text{v}}_{9}-0.13{\text{v}}_{16}+0.20{\text{v}}_{17}-0.21{\text{v}}_{18}-0.008{\text{v}}_{25}+1.01{\text{v}}_{33}$$

(m3G) $${\text{t}}_{\text{MCIcon}}=-10.84-1.64{\text{v}}_{10}-0.32{\text{v}}_{19}+0.10{\text{v}}_{24}+0.56{\text{v}}_{31}+0.89{\text{v}}_{35}$$

(m3S) $${\text{t}}_{\text{MCIcon}}=-1.84-1.28{\text{v}}_{10}+0.21{\text{v}}_{16}-0.31{\text{v}}_{19}-1.23{\text{v}}_{27}+1.49{\text{v}}_{35}$$

(m4G) $${\text{t}}_{\text{MCIcon}}=-14.40-0.32{\text{v}}_{1}-1.48{\text{v}}_{10}-0.60{\text{v}}_{15}-0.35{\text{v}}_{19}-0.12{\text{v}}_{24}+0.49{\text{v}}_{31}-0.30{\text{v}}_{32}+0.77{\text{v}}_{35}$$

(m4S) $${\text{t}}_{\text{MCIcon}}=-11.16-2.02{\text{v}}_{10}+0.22{\text{v}}_{16}+0.37{\text{v}}_{18}-0.73{\text{v}}_{19}+0.08{\text{v}}_{24}-1.24{\text{v}}_{27}+0.48{\text{v}}_{28}+1.22{\text{v}}_{35}$$

When assessed individually, some of the variables did not show significant difference between converters and non-converters. They, however, contributed to the model performance significantly as covariates (see Table 7 and 8). Our results show that the average value of some variables, such as v8 (List A Recog), v15 (RCFT Recog), v16 (Letter fluency) and v33 (Clock score), for HC, MCI and AD groups were HC>MCI>AD, as expected (Table 9). The group who converted from HC to MCI/AD or MCI to AD within 36 months had higher values than the non-converters but these differences were not statistically significant. These variables might be important for clinical diagnosis of AD patients, but any single feature alone is not predictive of the disease progression. However, when combined with other tests, individual feature can contribute to the multivariate models significantly and the feature sets are potentially useful as prognostic tests.

**Table 9:** Mean values of each variable for the different clinical groups.

Variable	HC	MCI	AD	HC convert to MCI/AD			MCI convert to AD		
				**Non_** **Converters**	**Converters**	**Pvalue** **(t,chisq)**	**Non_** **converters**	**Converters**	**Pvalue**

v1	28.84	26.20	19.10	28.96	28.23	<0.001	27.37	25.79	0.002
v2	12.90	6.51	3.24	13.24	9.84	<0.001	7.53	5.91	0.034
v3	11.38	3.79	1.02	11.80	7.10	<0.001	5.03	3.04	0.012
v4	0.92	0.31	0.09	0.07	0.27	<0.001	0.60	0.70	0.498
v5	60.55	38.30	26.56	61.35	51.23	<0.001	41.07	35.40	0.001
v6	0.87	-1.37	-2.21	0.95	-0.03	<0.001	-1.18	-1.81	0.001
v7	0.79	-1.62	-2.53	0.89	-0.05	<0.001	-1.38	-2.02	0.002
v8	0.09	-1.16	-1.90	0.12	0.21	0.269	-0.98	-1.22	0.247
v9	-0.21	1.16	2.14	-0.26	0.58	<0.001	0.72	1.65	0.001
v10	0.46	-1.21	-2.06	0.52	-0.10	0.000	-0.70	-1.52	<0.001
v11	-0.54	-1.49	-3.23	-0.49	-0.84	0.052	-1.42	-1.61	0.364
v12	-1.01	-0.78	-0.49	-1.00	-1.00	0.481	-0.76	-0.76	0.488
v13	0.48	-0.82	-1.92	0.50	0.06	0.019	-0.52	-1.13	0.015
v14	0.53	-1.02	-2.15	0.56	0.06	0.016	-0.58	-1.39	0.010
v15	0.31	-1.15	-2.91	0.33	0.34	0.490	-0.36	-1.88	<0.001
v16	12.04	10.04	7.32	12.17	11.06	0.066	9.23	9.57	0.338
v17	12.38	9.03	5.31	12.50	11.87	0.120	9.23	8.83	0.305
v18	12.13	8.25	4.46	12.33	9.71	0.000	8.87	7.57	0.054
v19	12.15	8.60	4.89	12.34	9.87	0.001	9.33	7.59	0.015
v20	0.74	0.18	-1.12	0.78	0.72	0.329	0.44	0.03	0.047
v21	0.72	0.27	-0.82	0.76	0.66	0.260	0.50	0.27	0.206
v22	12.00	11.11	9.10	12.11	12.06	0.465	10.73	11.09	0.285
V23	11.68	9.85	6.53	11.76	10.65	0.019	10.13	9.33	0.131
v24	108.33	105.92	100.84	108.46	107.32	0.227	103.47	107.53	0.072
v25	111.62	108.91	104.22	111.82	110.32	0.132	106.60	110.32	0.072
v26	-0.05	0.48	2.10	-0.03	0.08	0.280	0.63	0.38	0.237
v27	0.02	0.08	0.28	0.03	0.06	0.277	0.26	0.04	0.145
v28	0.06	0.94	4.57	0.02	0.64	0.035	0.90	1.09	0.362
v29	0.08	0.17	0.64	0.07	0.23	0.147	0.19	0.29	0.254
v30	-0.34	0.35	1.83	-0.36	0.13	0.004	0.31	0.39	0.424
v31	0.73	1.55	3.08	0.67	1.06	0.105	1.00	1.76	0.074
v32	-0.32	0.05	0.57	-0.34	-0.02	0.046	-0.10	0.02	0.299
V33	9.75	9.31	7.22	9.79	9.90	0.083	9.60	9.33	0.117
V34	43.12	40.48	36.32	43.31	41.84	0.111	38.40	41.79	0.076
v35	0.04	1.21	5.74	0.03	0.15	0.025	0.73	1.37	<0.001
v36	2.62	3.71	4.06	2.56	3.07	0.116	3.90	3.24	0.148
v37	4.33	4.94	4.98	4.28	4.20	0.428	4.97	4.52	0.238

Columns HC, MCI and AD are the mean values of each variable (cognitive test). The other columns are the means values for the groups of converters and non-converters from HC or from MCI with the t-test (chi-square test for variable 4, a dichotomous variable with values Pass and Fail) for the comparison between the 2 groups.

## Conclusion and discussion

The GA was good at finding high accuracy solutions but the reported models are not necessarily the absolute best solutions, as the spread of AUC values was about 0.05. These results are consistent with the general theory of GAs. The comparison with a stepwise algorithm available within R package showed that the GA selected variable sets that performed better for identifying solutions for prediction of progression from MCI to AD or for prediction of progression from a healthy status to MCI to AD when compared to the stepwise selection. This result emphasizes that the contribution of combined variable sets are more important than the contributions of individual variables for the model accuracy.

Multiple sets of neuropsychological variables were identified by GA to best predict disease progression. For predicting progression from being classified as healthy to MCI or AD, the measures more frequently selected by GA were those reflecting memory recall (v3, LMII), memory acquisition (v5, Total Learning) and a difficult version of semantic fluency (v18, Category Switching Total). When considered as combinations, the measures selected by the GA as a set to predict progression from healthy to MCI or AD included measure of general cognition (v1, MMSE), memory recall (v3, LMII), memory acquisition (v5, Total Learning) and semantic fluency (v18, Category Switching Total). For prediction of progression from MCI to AD, the GA selected a different set of measures. The measures variables recognition memory (v10, CVLT-II total Recognition d'), visual memory (v15, Rey Complex Figure recognition), semantic fluency (v19, Category Switching (switches)), executive function (v31, Stroop Colour errs) and clinical dementia rating (v35, CDR Sum of Boxes) were selected most frequently. When considered in terms of a set of measures, one of the best sets selected by the GA to predict progression from MCI to AD consisted of measures of general cognition (v1, MMSE), recognition memory (v10, CVLT-II Total Recognition d'), visual memory (v15, Rey Complex Figure recognition), semantic fluency (v19, D-KEFS Category Switching (switches)), IQ (v24, WAIS-III UK Pred Full Score IQ) and executive function (v31, Stroop Colours errs). Some other small variable sets selected by GA were effective for prediction of progression to AD from MCI. Importantly, larger sets of variables were not selected by the GA as being predictive of prediction for AD from MCI. As expected the GA showed that with increases in the number of neuropsychological measures the sets identified by the GA included increased error, weakening their predictive utility for predicting progression to MCI or AD.

This study applied GA for prediction of AD progression by combining the results of a large set of neuropsychological measures from the AIBL study that they had been selected for their sensitivity to cognitive impairment in both MCI and AD. *In silico *simulations with the limited data showed the potential of GA application in the neural science area. We have clearly demonstrated that the combination of the variables is superior in performance than the use of single significant variables for prediction of progression of disease. Integration of the neuropsychologists' interpretation and recommendation for the specific features (tests) is the next step for extension of this study. The developed algorithm will also be tested and adjusted with more data collected to improve the prediction models. One of the advantages of GA is that the fitness function can be designed to target specific research questions directly. The GA algorithm developed from this study was implemented as a general solution that can be extended to other prediction models.

## Competing interests

Authors have no competing interests relevant to this paper.

## Authors' contributions

PJ wrote the program code and did the initial data analyses, wrote the technical report which served the first draft of the paper. LV contributed to the program coding, validated the models, and participated in the writing. WW coordinated some team work, oversaw the data analysis and contributed to revising the paper. PM assisted with devising the cognitive battery used for the study, guided the data analysis direction from clinical point of view, participated the writing and revising drafts of the paper. GS assisted with devising the cognitive battery used for the study, revised the clinical translation of the data analysis and contributed to the writing and revising drafts of the paper. PG oversaw the data analyses and contributed to revising the paper. LM oversaw the data analyses, led the CSIRO team working on the AIBL study, served on the management committee, and contributed to revising drafts of the paper. KE coordinated the AIBL project, assisted with devising the cognitive battery used for the study and oversaw data collection, contributed the revision of the paper. CS, RNM, CCR, CLM and DA served on the AIBL management committee, involved with the initial conceptualisation of the AIBL research plan, and contributed to revision of the paper. PZ designed the methodology, supervised and participated in the data analyses, wrote the draft of the paper and contributed to its revision.

## Declaration

The publication charges were funded by the corresponding author's institution.

This article has been published as part of *BMC Bioinformatics *Volume 15 Supplement 16, 2014: Thirteenth International Conference on Bioinformatics (InCoB2014): Bioinformatics. The full contents of the supplement are available online at http://www.biomedcentral.com/bmcbioinformatics/supplements/15/S16.
